# Football Fans in Training: the development and optimization of an intervention delivered through professional sports clubs to help men lose weight, become more active and adopt healthier eating habits

**DOI:** 10.1186/1471-2458-13-232

**Published:** 2013-03-16

**Authors:** Cindy M Gray, Kate Hunt, Nanette Mutrie, Annie S Anderson, Jim Leishman, Lindsay Dalgarno, Sally Wyke

**Affiliations:** 1 Institute of Health and Wellbeing, 27 Bute Gardens, University of Glasgow, Glasgow, G12 8RS, UK; 2 MRC/CSO Social and Public Health Sciences Unit, 4 Lilybank Gardens, Glasgow, G12 8RZ, UK; 3 Sport, Physical Education and Health Sciences, St Leonard’s Land, University of Edinburgh, Holyrood Road, Edinburgh, EH8 8AQ, UK; 4 Centre for Public Health Nutrition Research, University of Dundee, Dundee, DD2 4BF, UK; 5 NHS Forth Valley, Falkirk, FK1 4PP, UK

## Abstract

**Background:**

The prevalence of obesity in men is rising, but they are less likely than women to engage in existing weight management programmes. The potential of professional sports club settings to engage men in health promotion activities is being increasingly recognised. This paper describes the development and optimization of the Football Fans in Training (FFIT) programme, which aims to help overweight men (many of them football supporters) lose weight through becoming more active and adopting healthier eating habits.

**Methods:**

The MRC Framework for the design and evaluation of complex interventions was used to guide programme development in two phases. In Phase 1, a multidisciplinary working group developed the pilot programme (p-FFIT) and used a scoping review to summarize previous research and identify the target population. Phase 2 involved a process evaluation of p-FFIT in 11 Scottish Premier League (SPL) clubs. Participant and coach feedback, focus group discussions and interviews explored the utility/acceptability of programme components and suggestions for changes. Programme session observations identified examples of good practice and problems/issues with delivery. Together, these findings informed redevelopment of the optimized programme (FFIT), whose components were mapped onto specific behaviour change techniques using an evidence-based taxonomy.

**Results:**

p-FFIT comprised 12, weekly, gender-sensitised, group-based weight management classroom and ‘pitch-side’ physical activity sessions. These in-stadia sessions were complemented by an incremental, pedometer-based walking programme. p-FFIT was targeted at men aged 35-65 years with body mass index ≥ 27 kg/m^2^. Phase 2 demonstrated that participants in p-FFIT were enthusiastic about both the classroom and physical activity components, and valued the camaraderie and peer-support offered by the programme. Coaches appreciated the simplicity of the key healthy eating and physical activity messages. Suggestions for improvements that were incorporated into the optimized FFIT programme included: more varied in-stadia physical activity with football-related components; post-programme weight management support (emails and a reunion session); and additional training for coaches in SMART goal setting and the pedometer-based walking programme.

**Conclusions:**

The Football Fans in Training programme is highly acceptable to participants and SPL coaches, and is appropriate for evaluation in a randomised controlled trial.

## Background

The prevalence of obesity in men in the UK is among the highest in Europe [1,2] and is forecast to increase at a faster rate than female obesity in the next 35 years [3]. Excess weight is associated with an increased risk of poor health and premature mortality. Obese men are three times as likely to have high blood pressure as men of normal weight [4]; 90% of cases of type 2 diabetes can be attributed to excess weight; and there is a more than twofold increase in the risk of coronary artery disease and stroke amongst obese people [3]. After smoking, obesity is considered the most important preventable cause of cancer [4]: every additional 5 kg/m^2^ in body mass index (BMI) increases a man’s risk of oesophageal cancer by 52%, thyroid cancer by 33%, and colon and renal cancer by 24% [5].

Men’s lifestyles make a significant contribution to gender inequalities in health [6]. Many men have poor diets, with a low intake of fruit and vegetables and a high intake of fat [7], and tend to drink more alcohol than women [8-10]. The majority also fail to meet national guidelines for physical activity and health [2]. In addition, men appear more reluctant than women to engage in existing weight management and other programmes aimed at encouraging people to live healthier lifestyles [11-16]. However, evidence suggests that when gender issues are used to inform programme design and delivery, men *will* engage with weight management initiatives [17-20], although there have been few high quality randomised control trials of male-only interventions [15,18].

Recently, the potential of professional sports organisations to attract men to participate in a range of health promotion initiatives has been recognised [21-27]. Sports clubs can maximise engagement by capitalising on the traditional male sporting environment and the powerful social and psychological connections to the team (e.g., loyalty, identity, validation, belonging) that ‘being a fan’ creates [28]. Pilot initiatives have begun to explore the possibility of delivering weight management advice to men through professional sports clubs [24,27]. For example, forty men taking part in a men’s health initiative at Celtic and Rangers Football Clubs in Glasgow achieved an average 4% weight loss during a 10-week programme and continued to lose weight over the following 12 months [27].

Previous research into delivering health promotion through professional sports club settings has been small scale and/or has lacked scientific rigour. This has led to a strong recommendation for rigorous, controlled evaluations to be conducted in this area [29,30]. MRC guidance for developing and evaluating complex interventions advises that in order to conduct a high quality evaluation with potential for maximum impact, a systematic approach to intervention development should be followed [31]. This phased approach includes the iterative processes of: identification of the evidence base; developing a theoretical understanding; and using pilot work (which considers the views of the target population, those delivering the programme and the practicalities of implementation) to inform final modifications to the programme prior to full-scale evaluation.

Following this framework, this paper describes the development and optimization of the Football Fans in Training (FFIT) programme which aims to use the draw of professional football clubs to engage overweight and obese men in weight loss, physical activity and healthy eating.

## Methods

**Figure 1:**
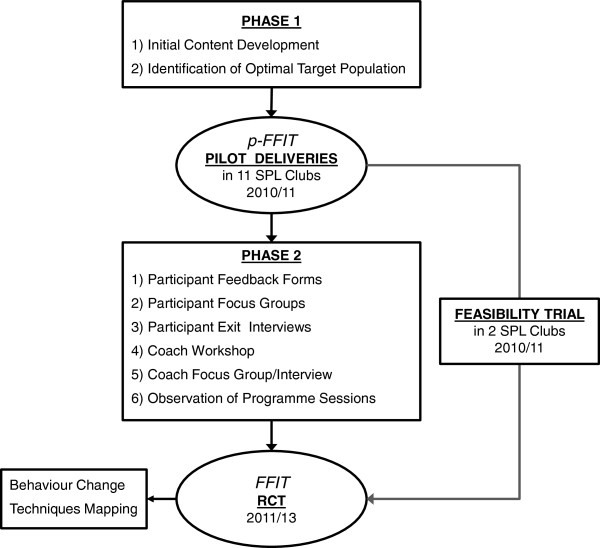
Overview of FFIT programme development and optimization.

FFIT was designed in two phases. Figure 1 provides a schematic overview of the processes involved.

### Phase 1 programme development

Phase 1 consisted of two steps. In step 1, the development of the pilot programme (p-FFIT) was led by an expert multidisciplinary working group comprising: two psychologists (one an exercise psychologist); two health social scientists (one with expertise in gender and health); a nutritionist; a men’s health nurse (with expertise in weight management for men); and a representative from the Scottish Premier League (SPL) Trust (which has a remit to deliver social change through community engagement within SPL clubs) [32]. The group met formally on two occasions and worked iteratively via email or in smaller sub-groups on successive drafts of the programme. In step 2, the optimal target population was identified by conducting a scoping review [33] to summarise existing evidence on men’s motivation to lose weight and improve their lifestyles (e.g., pressure from family members, wanting to be able to do more with their children, personal and family health histories), the potential health benefits of weight loss and increased physical activity, and current weight management and physical activity guidance.

### Phase 2 programme optimization

Phase 2 consisted of 3 steps: process evaluation; programme redevelopment; and mapping of behaviour change techniques. In step 1, p-FFIT was piloted in 11 of Scotland’s top professional SPL football clubs in two deliveries: the first in autumn 2010 (Delivery 1); the second in spring 2011 (Delivery 2). Most clubs recruited participants by advertising the programme on their websites; a few used also used loudspeaker announcements at home matches. At most clubs the programme was delivered by male community coaches who had a range of qualifications and were employed by the clubs to deliver their community (usually football-based) activities; a female coach supported her male colleague in one club, and a few clubs engaged external male health trainers to support their staff in programme delivery. A process evaluation explored programme delivery from both participant and coach viewpoints, and included observation of session deliveries. At the same time, a feasibility trial was conducted in two of the clubs to investigate recruitment, retention and potential weight loss in preparation for a subsequent randomised controlled trial. The results of the feasibility trial are available elsewhere [34,35]. Ethical permission was obtained from the School of Nursing, Midwifery and Health at the University of Stirling.

As part of an SPL Trust audit of Delivery 1 (conducted as part of the reporting to the Scottish Government and Football Pools, who funded the delivery of p-FFIT), men completing the programme across all participating clubs were asked to fill out feedback forms. The feedback forms consisted of open questions to identify what participants liked about the programme and to elicit suggestions for improvements or additions. The feedback forms were anonymous to encourage the men to be open and honest; however, this meant it was not possible to examine differences in opinions according to outcomes. All responses were read through to identify the key themes, including: the main elements men liked about p-FFIT; suggested improvements; and suggested additions. A matrix (with rows representing responses and columns representing themes) was then used to identify occurrences of each theme to allow frequency analyses to be performed.

Focus group discussions with a sample of men who completed p-FFIT (sampled purposively from a list of volunteers to represent the range of ages and baseline BMIs) and telephone or face-to-face interviews with non-completers were conducted at the two clubs participating in the feasibility trial. Those delivering p-FFIT at these two clubs were interviewed using a focus group discussion at one club (three community coaches and one men’s health trainer, all male) and a paired interview (one community coach and one health trainer, both male) at the other club. We used a semi-structured format for all focus group discussions and interviews to explore: the acceptability of/satisfaction with p-FFIT; components that were useful/not useful; and suggestions for changes. In addition, community coaches from all clubs (all male) attended a FFIT feedback and training workshop midway through Delivery 1.

The focus group discussions, interviews and workshop were audio-recorded with participants’ consent and transcribed verbatim. Transcripts were analysed thematically using the Framework Approach [36], and NVivo9 software was used to assist data coding and organisation. The coding frame was based on our main research questions (concerning acceptability/satisfaction, views on likely effectiveness of programme components and suggestions for changes), but also allowed unanticipated themes to emerge and be systematically explored. Summary analyses of four key themes are relevant here: *Group factors*, which included any reference to group dynamics and interaction with other group members; *Programme components*, which included references to elements that participants found useful/not useful in losing weight or becoming more active; *Points for future consideration*, which included specific issues and recommendations; and *Exit reasons*, which captured reasons participants gave for non-completion of p-FFIT. All four themes provided information that was relevant to the acceptability of the programme to participants and coaches, and data to inform programme optimization. A subsample of transcripts (n = 3) was cross-coded to verify high consistency of coding.

Extracts from the focus group discussions and interviews are labelled to indicate the source (“PFG1” = Delivery 1 participant focus group; “PFG2” = Delivery 2 participant focus group; “PExit” = interview with man who did not complete p-FFIT in Deliveries 1 and 2; “CFG” = coach focus group; “CInt” = coach interview; “CWorkshop” = coach workshop) and participant or coach ID.

During the observations (conducted by CMG), programme sessions were audio-recorded, and detailed notes taken with the consent of the coaches and participants according to session-specific observation proformas that were developed to reflect the detailed content of each weekly session. Notes were subsequently written up electronically. The observation proformas (an example of which is provided in Table 1) focused on: the extent to which coaches adhered to the p-FFIT delivery protocol for each session; group-based factors; and identification of examples of particularly good practice and/or problems/issues.

**Table 1:** Observation proforma for p-FFIT week 2

**SPL FFIT Session 2 what are we eating?**	**Field notes**	
Date, time and venue:		
Coaches:		
No. of men and layout of room:		
**Session delivery protocol/recommended timings**		
**1. Welcome back/2 minutes**		
A warm welcome for making it to the second session can be a great boost to men’s confidence and feeling of self-worth. People feel valued if you acknowledge their presence. Important to start on time and encourage all men to interact from the outset.		
**2. Food diaries/5 minutes**		
Immediately raising the homework gives people a sense that the work starts now! Invite the men to say a bit about how the food diary homework went. Was it easy or difficult? Did they eat what they thought they ate or were there any surprises?	.	
**3. Eating well/25 minutes**		
**Preparation: The Eatwell plate mat with a display of foods representing each of the food groups should be prepared beforehand.**		
Use the Eatwell display to talk through the concept of the five food groups and the different proportions from each group that make up a healthy diet. Take time to illustrate both the proportions recommended for each food group and the portion sizes for the foods represented. Discuss how this compares to what they would normally eat. Now, ask each person to look at their own food diary and write down the number of portions they had from each food group. Then encourage the men to discuss the results as a group. Were there any surprises? Did they notice which foods they ate too much of, and which they ate too little of?		
**4. Setting goals/10 minutes**		
Ask the men, in pairs, to think about their food diaries and each write down two goals that would help them achieve a more balanced diet. Stress their goals should be SMART (specific, measurable and achievable, realistic and time limited), give examples of SMART goals, and warn against setting huge, unrealistic goals that will only set them up to fail.		
Check that everyone has set two goals and convey your optimism to the group that they *will* succeed in meeting their goals.		
**5. Pedometer steps/20 minutes**		
Moving on from the food-based discussion to a review of the men’s physical activity helps to reinforce the link between the two major aspects of the programme – “eating a healthier, more balanced diet” and “being active”. Refer to the men’s “Baseline steps” homework and ask the men how they found recording their daily steps. Explain that while how much we walk is dependent on a variety of factors, in general if you walk less than 5,000 steps a day this is seen as fairly sedentary, while if you record more than 10,000 steps this is seen as quite active. Ask the men to set a goal related to physical activity over the next week. Suggest that this may involve increasing their steps by an extra 1500 a day on three days of the week, and make sure they know where to record this. Discuss tips for increasing walking.	.	
**6. Active session/28 minutes**		
Consider another walk in the stadium (e.g., round the pitch)		

In step 2, the findings from the participant feedback forms, the participant and coach focus group discussions and interviews, the coach workshop and the session observations were triangulated to produce a detailed description to inform the redevelopment of the optimized programme (FFIT). Finally, in step 3, two members of the programme development working group (CMG and SW) used Michie and colleagues’ BCT Taxonomy v1 [37] to map the content of FFIT onto specific behaviour change techniques. Initial coding was carried out independently by each coder using NVivo9 software. Subsequent comparison and discussion of discrepancies produced 100% agreement.

## Results

### Phase 1 programme development

Step 1 led to the development of p-FFIT, comprising 12, weekly, 90-minute, gender-sensitised, group-based classroom and physical activity sessions. The programme was designed to be delivered by SPL community coaches in club stadia at no cost to participants^a^. The delivery protocol was closely based on the ‘Camelon’ men’s weight management model, which was developed for delivery to men in a National Health Service (NHS) setting [17], but was extended to include greater emphasis on physical activity by drawing on evidence for increasing physical activity in inactive people [38]. This included provision of a pedometer to enable self-monitoring of walking (as incremental, pedometer-based daily step count targets have been shown to increase step counts for adults in community-based interventions [39]), setting achievable goals to build confidence and motivation, and exploring ways of finding social support.

The programme development working group ensured that p-FFIT adhered to current national guidance for weight management programmes [40,41]. Links were made to current websites [42,43] to provide additional dietary and physical activity advice and support. A participant information booklet (including tables to record self-monitored weight loss and daily step counts) and detailed delivery notes for coaches were developed. Coaches’ notes were supplemented by one and a half days of group-based training from members of the programme development working group in nutrition, physical activity and behaviour change techniques. All men enrolling in p-FFIT completed a Physical Activity Readiness Questionnaire (PAR-Q) [44] to identify any contraindications to exercise. Those answering ‘Yes’ to any question were required to produce a letter of support from their GP before being accepted onto the programme.

Step 2 identified the target group most likely to benefit, as men aged 35-65 years with a body mass index (BMI) ≥ 27 kg/m^2^. The lower age limit reflects evidence that overweight and obese men in their 30s may experience an attitudinal shift in relation to their health and physical limitations as they approach middle age [45]. This attitudinal shift means that men in this age group are likely to be more receptive to advice on changing health behaviours than younger men, thus increasing the potential effectiveness of lifestyle interventions [3]. The upper age limit reflects differences in physical activity guidelines for over-65 s [46], and the fact that the complexity of associated health problems in older age groups reduces the potential for public health gain. The BMI cut off reflects findings that men who are obese, or at high risk of becoming obese, are more likely to want to lose weight than those who just exceed the normal weight range [17,47]. Clubs were given the target of recruiting 30 men to each delivery. However, as evidence from the ‘Camelon’ model [17] suggested that the maximum group size should be 15 men, coaches were asked to split each delivery cohort into two smaller groups.

### Phase 2 programme optimization

#### Step 1 – process evaluation

Of the 303 men who took part in Delivery 1 across 11 SPL clubs, 155 (51.2%) returned anonymous post-programme feedback forms. At the two clubs involved in the feasibility trial, 26 men who completed p-FFIT joined focus group discussions (two conducted in December 2010 with participants from Delivery 1 and two conducted in April 2011 with Delivery 2 participants); and a further 13 men who did not complete the programme underwent ‘exit’ interviews in December 2010 and April 2011. Coaches delivering p-FFIT at these two clubs took part in a focus group discussion (n = 4) or face-to-face interview (n = 2) following Delivery 1. Coaches from all 11 clubs attended the FFIT workshop in October 2010.

Seven programme sessions were observed across p-FFIT Deliveries 1 and 2 at each of the two clubs involved in the feasibility trial; one session was observed at all other clubs during Delivery 2. The observation schedule ensured that all 12 programme sessions were observed across the clubs.

#### Group factors

The participants and coaches were extremely positive about p-FFIT. Men responding to the open-ended questions on the feedback forms (see Table 2) highlighted the camaraderie and the friendly, relaxed, non-directive manner in which the programme was delivered. Men taking part in the focus group discussions described how the group setting and the fact that they perceived other members to be similar to themselves had helped foster peer support:

"PFG1 125: I think it was the banter and the shared experience and folk coming back and saying, “I couldnae do as many steps this week”, but somebody saying, ”Oh, I took the dog out and…”, you know, it was just all shared ideas and experiences that really worked."

"PFG1 244: It’s not just the guys with the same type of interest, but the age grouping was a good idea as well; and the fact that there wasn’t going to be any Greek gods in there, it was all going to be human beings, cherubs perhaps, so you’re not going to feel out of place."

**Table 2:** Participant feedback (percentages of men spontaneously mentioning each factor in response to open-ended questions)

Liked (N = 154)*		**Improvements (N = 78)**		**Additions (N = 63)**	
Camaraderie	55.8(86)	More physical activity, less theory	26.9(21)	More football	33.3(21)
Physical activity	35.7(55)	Better facilities	10.3(8)	More/different types of physical activity	20.6(13)
Lifestyle education (eating/alcohol)	29.2(45)	More football	9.0(7)	Continuation/follow up beyond 12 weeks	12.7(8)
Coaches (friendly, treated as an adult)	20.1(31)	Different timing (summer/weekend)	9.0(7)	Liaising with other FFIT groups	9.5(6)
Association with club	14.9(23)	Coaches being better organised	7.7(6)	Health checks	7.9(5)
Booklet/information/structured approach	14.3(22)	More club involvement	7.7(6)	Meeting players/coaches/managers	7.9(5)
Being motivated to improve	13.6(21)	Club T-shirts arriving on time/at all	6.4(5)	Individualised physical activity	7.9(5)
Informal environment	9.1(14)	Continuation/follow up sessions	6.4(5)	Healthy eating suggestions/nutritionist	6.3(4)
5-a-side football matches	7.1(11)	More than one night per week	5.1(4)		
No pressure – advice, not orders	5.8(9)	More individualisation of physical activity	5.1(4)		
Outcomes (losing weight/getting fit)	5.2(8)	More detailed information to supplement booklet	3.8(3)		
Pedometer/walking	4.5(7)	Varied physical activity/trips to local facilities	2.6(2)		
Timing/weekly meeting/time of year	4.5(7)	Need for commitment stressed more	2.6(2)		
Meeting players/guest speakers	3.9(6)	Discount for local facilities	2.6(2)		
Entrance requirements	3.2(5)	Shorter weigh-ins	2.6(2)		
Targets (reviewed regularly/achievable)	3.2(5)	More one-to-one time	2.6(2)		
Finding it was easy to change	1.9(3)				

*number of participants providing a response shown in brackets.

Practical constraints meant that some clubs were unable to restrict their group size to 15 men. Session observations suggested that the larger group sizes worked well on the whole, particularly during the physical activity sessions, where there was a spirit of teamwork and co-operation. However, some participants and coaches highlighted difficulties in raising sensitive issues in the group setting:

"PExit 151: I was sorry I couldn’t participate in the physical exercises they did, but I didn’t want to get embarrassed and be out of puff and look like an idiot, grunting away there. (Interviewer: Do you think they [coaches] could have done more to accommodate you?) I didn’t really, no. I mean, I don’t blame them for that at all. No, no, no, I just didn’t want to bring it up."

"CFG 22: If we were walking over to the gym, it would be kind of get feedback that way… because rather than standing up, I think sometimes as a group, some people might no’ speak up but they’re wanting to tell you stuff, so […] I walked at the back and [if] one of them seen me he would maybe come and just have a wee blether [chat]. So it was good that way because I was able to speak to them more one-to-one just to see how they were feeling, get a wee bit of feedback to them."

One man felt more could have been done to foster a sense of belonging:

"PFG1 131: I was kind of struggling every week to remember folks’ names and it would have been good to have embedded that a little bit more at the beginning of the programme so the guys could have gotten to know each other."

#### Programme components

Men completing the post-programme feedback forms also highlighted the in-stadia physical activity sessions. Broad guidance was given in the programme notes as to what each activity session should aim to deliver (i.e., 20-30 minutes of incremental cardiovascular, strength and flexibility exercises designed to accommodate different levels of fitness and ability), but the coaches were encouraged to adapt this guidance to suit group preferences and available facilities. A number (but not all) of the clubs had incorporated small-sided football games, and these were appreciated. Participants were also positive about the pedometer-based walking programme (for more detailed discussion of men’s experiences of the pedometer programme, see reference [48]) and the SPL club setting.

In the classroom, participants liked the fact that p-FFIT provided an overall lifestyle education rather than taking a diet-focussed approach to weight loss. Focus group participants highlighted the information about portion sizes (delivered in week 2, see Table 3) and food labelling (week 9) as being particularly useful:

"PExit 243: It was when they showed us the portion sizes that you should be eating, that was the real shocker for me because, you know, even if you’re eating fairly healthily, the amount that I was eating wasn’t doing me any favours."

"PFG2 128: I just couldn’t believe… some of the stuff I thought I was taking [eating] was okay, and when we did the bit on labels and stuff, I was checking some of the things that I used to have and thinking, “My God, that’s got about three days worth of sugar in it, and I’ve had it in one meal!”"

**Table 3:** FFIT key components*

	**Classroom**	**Physical activity**
**week 1**	· Programme overview	· Introduction to pedometers
	· Need for commitment	· Baseline step count
	· Personal responsibility and perceptions of control over eating	· Short stadium tour wearing pedometer
	· Food diary	
**week 2**	· Healthier eating and portion sizes using Eatwell Plate	· Setting progressive, individual step count targets
	· SMART goal setting for eating	· Brisk walk around the pitch
**week 3**	· Goal review§	· Step count review§
	· Individual eating plans (600 kcal/day deficit)	· Principles of fitness: warm up, aerobic, strength, flexibility, cool down
	· Health benefits associated with weight loss	· Warm up activities
	· Role of social support	
**week 4**	· Importance of physical activity to health and wellbeing	· Heart rate monitoring and Rating of Perceived Exertion (RPE) Scale
	· Barriers to being active	· Warm up and 20-minute aerobic workout
	· Action planning for physical activity	
**week 5**	· Measuring alcohol units	· Warm up and 20/30-minute aerobic workout
	· Alcohol, other drinks and weight gain	
	· Planning your drinking	
**week 6**	· Formal weigh-in to review progress	· Principles of strength training using own body weight
	· Relapse prevention	· Warm up, aerobic and strength training
	· Role model for inspiration	
**week 7**	· Physical representation of weight loss achieved (sandbags)	· Principles of stretching and flexibility
	· Motivation and confidence	· Warm up, aerobic and strength training with flexibility component at end
	· Reflection on progress	
**week 8**	· Understanding food packaging labels	· Circuit of aerobic and strength activities with warm up/cool down
	· Importance of breakfast and regular meals	
**week 9**	· Making favourite meals healthier	· Similar circuit to week 8
	· Making eating out/takeaway meals healthier	
**week 10**	· Common perceptions about healthy living	· Visit to local physical activity facilities or circuit of warm up, aerobic, strength and flexibility activities
	· Links between emotions and behaviour	
	· Relapse prevention	
	· Food diary	
**week 11**	· Review of progress made in eating, physical activity and locus of control	· Visit to local physical activity facilities or circuit of warm up, aerobic, strength and flexibility activities
	· The energy balance	
**week 12**	· Formal weigh-in, and personal and group feedback	· Circuit of activities or, if appropriate, small sided soccer games
	· Relapse prevention	
	· Ongoing social support	
**Reunion**	· Review of experiences in maintaining dietary and physical activity changes	· Circuit of activities similar to those provided towards the end of the 12-week ‘weight-loss’ phase
	· SMART goal setting for 12 months	
	· Ongoing social support	

* Attendance is taken and participants are encouraged to record their weight at the start of each session.

§ SMART goals and step counts are reviewed regularly from week 3.

The coaches felt a major strength of p-FFIT was that the key messages were easy to understand:

"CInt 12: I’m quite a big fan of the Eatwell Plate and just how it works; it simplifies it for a lot of people. And that, and I think the portions, together I think is really, really good […]. The alcohol element as well is also one that folk don’t really realise what a measure is or how many calories are in certain things. So I think they were certainly very important parts."

The physical representation of midpoint weight loss (in week 7 coaches are asked to use sandbags to illustrate group and individual weight loss) proved to be a powerful motivator, even for men who were less successful at losing weight at this stage:

"PFG2 205: I thought that was thoroughly good because there was one person in the group, we’ll no name anybody, had a bag full, and I thought, “Look at that bag”, and then I looked at mine, and I went, “Hey, wait a minute here!” And that guy actually pushed me to say “Right, I’m going to go even harder now” […] and the last five weeks, bang, as if everything just dropped off."

In contrast, observation of the eating plan session (week 3) in two clubs indicated that both participants and coaches experienced some difficulties in calculating the daily calorie intake for weight loss. Focus group participants confirmed they had found this component less useful:

"PFG2 126: I don’t know what it was about that session, my eyes glazed over when that was going on and I thought, “Bugger this, I’m not going to do that” […] I’m sitting there thinking, “This is too much like hard work for me”."

#### Points for future consideration

Observation of the classroom sessions demonstrated that fidelity to the p-FFIT delivery protocol was, on the whole, good. However, some coaches admitted it had been difficult to find sufficient time to read through and assimilate the detailed delivery notes in preparation for each session:

"CWorkshop 31: The first two weeks, no excuses, I was up to here with everything else work-wise, and didn’t really read it thoroughly enough. So I was at the start and just looked at the content [pages] instead of looking further in… so we made up our own."

There was a tendency for some coaches to read directly from the notes, and delivery of key points was sometimes rushed. The ‘classroom’ part of the sessions (particularly the more information-rich early sessions) often over-ran and encroached on the time available for group physical activity:

"CFG 24: I had to wait until [Coach 23] was finished [the classroom delivery]… I was only going to get 25 to 30 minutes of activity, and for once a week that's not enough. So I needed more time, I was needing more time, but I couldn’t get it because of the timescale we had, because you had to do the education stuff."

The in-stadia physical activity sessions did not always adhere strictly to the guidance provided: in some clubs the same activities were offered each week. Nevertheless, most coaches appeared skilled at encouraging participants to work at a level of intensity that was appropriate for their individual fitness and ability. The main exception was during small-sided football games, where some men appeared to push themselves too far. Participant feedback (shown in Table 2) confirmed that many of the men wanted more time and more variety during the physical activity sessions, as well as more emphasis on football-related activities.

Although participants appeared to embrace goal-setting, the session observations showed that coaches did not always ensure that goals were SMART (Specific, Measureable, Achievable, Recorded, Time-limited): some lacked specificity, were over-ambitious or were not time-limited. There was also some confusion over the pedometer-based walking programme, with uncertainty about whether activities other than walking could count towards daily step targets, and whether the baseline step count should remain the same throughout the programme or increase each week. This led to some participants setting inflated step count targets, which they found demotivating:

"PExit 102: I felt guilty because I didn’t want to… hold anybody else back, and the other guys that were in the course were really motivated and, you know, basically from what I could see, they had the time to do the necessary stuff. I just didn’t and I just felt like I was letting people down because I hadn’t done my step count that week."

Whilst participants felt that being shown a physical representation of weight loss in week 7 was highly motivational, some coaches found it difficult to supply sandbags as recommended. This session was observed in two clubs, and neither succeeded in accurately representing both whole group and individual weight loss. Coaches in one club used gym weights to demonstrate whole group weight loss only; the other club used sand-filled padded envelopes to illustrate individual weight loss, but did not provide enough envelopes to represent whole group weight loss.

Other issues included men who had answered ‘Yes’ to questions on the PAR-Q being unable to take up their place on p-FFIT because their GP had been reluctant to support their involvement:

"CWorkshop, 41: It wasn’t that people didn’t want to go on the programme. I think a few [clubs] have probably experienced it. I know I spoke to [the SPL Trust] about it, and the GP had not signed the letter for a couple of the boys, so they’re still hanging in there just now [waiting to get on the programme]."

The lack of provision of post-programme follow-up was also raised by both participants and coaches:

"CWorkshop 51: We even talked about charging them [for additional sessions after the end of p-FFIT] so they could come along and do it, because I think that whole thing of being clubs, individual clubs doing it, brings that unitedness in doing something together."

#### Exit reasons

The exit interviews showed that most men who did not complete p-FFIT left because of reasons that were unrelated to the programme. Work commitments (n = 3) and health issues (n = 3) were the most common reasons for non-completion. Others included: moving away from the area (n = 2), family commitments (n = 2) and bereavement (n = 1). However, one man cited lack of variety in the physical activity sessions as one of the reasons he stopped attending.

### Step 2 – programme redevelopment

The process evaluation confirmed that p-FFIT was highly acceptable to both participants and coaches. However, a number of potential areas for improvement were identified. Where possible, these were incorporated into the optimized FFIT intervention.

#### Optimizing the group setting

The maximum group size guidance has been replaced by a recommended coach:participant ratio of at least 1:15. This reflects operational constraints at some clubs whilst ensuring sufficient staff capacity to deliver one-to-one support.

Guidance to coaches is modified to encourage them to take a formal register at the start of each weekly session to promote familiarity among group members.

The BMI inclusion criterion has been raised to ≥ 28 kg/m^2^ to further foster camaraderie and a sense of belonging. This reflects the finding that participants feel most comfortable with others they perceive to be similar to themselves, in terms of goals as well as appearance (some men with lower BMIs seemed more interested in achieving ‘fitness’ than increasing their daily activity, and were also less focused on losing weight).

#### Optimizing the classroom components

The delivery notes have been simplified, and a bullet-point list added to emphasise the key components of each session and to encourage coaches not to read directly from the booklet during programme delivery. Components that could be omitted if time runs short have been identified to give coaches strategies to alleviate time pressure if discussion of the key content runs on.

A list of essential preparation is provided for each session to encourage coaches to be organised.

An online tool^b^ has been developed to assist coaches and participants in calculating daily calorie intake for weight loss (week 3).

Advice is given that gym weights or other equipment can be used to represent weight loss instead of sandbags (week 7), but the importance of representing both group and individual weight loss is stressed.

The food labels session has been moved forward from week 9 to week 8, as many men appear to find this information useful and may benefit from receiving it earlier.

#### Optimizing the physical activity components

Information about how activities other than walking (e.g., swimming) can contribute to the daily step count target has been provided.

A more detailed protocol for the physical activity sessions encourages more variety and football-based activities (e.g., football training drills).

#### Other changes

Post-programme support: In response to concerns from both participants and coaches about lack of follow-up, two weight maintenance components have been added following the initial 12, weekly ‘weight loss’ sessions:

1. Six standardised email prompts reinforcing key messages have been developed to be sent out by the coaches at specific time points in the 9 months following the end of the 12-week ‘weight loss’ phase. Their content emphasises self-monitoring, goal setting and relapse prevention.

2. A reunion session at the club 6 months after the end of the 12-week ‘weight loss’ phase encourages men to discuss their experiences of maintenance of weight loss, and of physical activity and dietary change.

Training: Two days of coach training have been developed to include more emphasis on SMART goal setting and the pedometer-based walking programme. The training is highly interactive and designed to promote the principles of adult learning [acting as a facilitator, encouraging mutual respect, and building on life experiences and existing knowledge [49]] and the use of banter in the group sessions. Coaches are also encouraged to share ideas about how to develop a varied and individualised in-stadia physical activity programme.

Enrolment: The requirement for men answering ‘Yes’ to questions on the PAR-Q to provide a GP letter endorsing their participation has been dropped. Instead, the coaches simply advise these men to speak to their GP before commencing the physical activity components of the programme. In addition, to ensure participant safety, the coaches now measure blood pressure at enrolment. Any man who exceeds 159 mmHg systolic or 99 mmHg diastolic is encouraged to take part in the classroom sessions and pedometer-based walking programme, but is excluded from more vigorous in-stadia training until he provides evidence that his blood pressure has reduced.

#### The optimized FFIT programme

FFIT is a group-based, weight management, physical activity and healthy eating programme consisting of an initial intensive ‘weight loss’ phase (12, weekly, 90-minute sessions delivered free of charge to participants at football stadia by club community coaches) and ongoing ‘light touch’ weight maintenance support to 12 months. The dietary component of FFIT is designed to deliver a 600 kcal daily deficit (from estimated daily energy requirements) [40,41] through: the gradual adoption of nutrient-dense foods and reduction of the portion size of energy-dense foods; and the reduction of sugary and alcoholic drinks. Classroom activities are aimed at encouraging participants to make dietary changes that suit their individual eating preferences, to weigh themselves each week and to keep a personal record of their weekly weight loss.

FFIT has two physical activity components: First, the incremental pedometer-based walking programme [39,50] encourages men to set individual daily brisk walking goals to include more walking in their daily routine and to report their progress to the group each week. Men able to do more vigorous physical activity are encouraged to supplement their walking with additional exercise (e.g., gym sessions), and to count this toward their daily steps target.

Second, in-stadia physical activity sessions teach participants how to build fitness through structured activities that are tailor-able to individual fitness levels and ability, and include aerobic, muscle strengthening and flexibility exercises [38]. Men are also encouraged to avoid compensatory behaviours (e.g., increased snacking or television viewing) which can undermine weight loss following exercise [51,52], and to meet in between programme sessions to exercise together (e.g., walking, cycling or using local sports facilities). The key components of the classroom and in-stadia physical activity sessions are summarised in Table 3.

A number of components are specifically designed to appeal to male football fans. These include: club-based incentives (e.g., club T-shirts, visits from club celebrities); elements of competition (e.g., through quizzes); an entire classroom session (week 5) devoted to discussion of the role of alcohol in weight gain and strategies for reducing alcohol consumption; and the use of ‘banter’ to facilitate men’s discussions of sensitive issues, such as weight gain [53,54].

Ongoing support is provided after the end of the 12-week ‘weight-loss’ phase through six email prompts, which are sent out by club coaches at 6-weekly intervals, and one reunion session at the club. Men are also encouraged to continue to meet regularly to exercise together and to provide mutual support. These meetings can either be run by the club (some offer weekly physical activity sessions for a small cost) or organised independently using local sports facilities.

### Step 3 – mapping to behaviour change techniques

Mapping the content of FFIT onto Michie and colleagues’ BCT Taxonomy v1 [37] demonstrated that 37 specific behaviour change techniques are used throughout the programme. As shown in Table 4, FFIT draws heavily on self-monitoring, implementation intentions, goal setting and review, and feedback on behaviour, all of which are associated with control theory [55] and have been shown to be effective in physical activity and healthy eating interventions [56,57]. The programme also encourages social support, which has been shown to be effective in weight loss interventions [57]. Further key techniques used in FFIT draw from other theoretical accounts of behaviour change [e.g., social cognitive theory [58]] and include: information on consequences; identification of barriers to change; verbal persuasion about capability; instruction in performing new behaviours; graded tasks; and social comparison.

**Table 4:** Mapping between behaviour change techniques and FFIT programme sessions

**Label**	**Definition**	**Sessions**	**An example of how it is operationalized in FFIT**
***Social support***			
Social support (unspecified)	Advise on, arrange or provide social support or non-contingent praise or reward for the behaviour	week 1-7,10,12, email 2-4, reunion	The coach points out that if the men haven’t done so already, they should meet up outside the group (e.g., a walking group) – week 3
***Regulation***			
Reduce negative emotions	Advise on ways of reducing negative emotions to facilitate performance of the behaviour	week 4	The coach asks the men in their teams to come up with 5 barriers to physical activity (including not feeling like going out or that they can’t be bothered). Taking one barrier at a time, the coach asks the whole group to suggest how to overcome it – week 4
***Feedback and monitoring***			
Feedback on behaviour	Provide feedback on performance of the behaviour	week 2-6,9-12, reunion	Comparing their food diaries with the healthy eating plate can help the men understand the type of changes they may need for a healthier diet (i.e., smaller portions) – week 2
Feedback on outcome(s) of behaviour	Provide feedback on the outcome of performance of the behaviour	week 6,7,12	The coach takes each man individually to record his weight and waist measurement and tell him how much weight he has lost – week 6
Self-monitoring of behaviour	Establish a method for the person to monitor and record the behaviour(s) as part of a behaviour change strategy	week 1-12, email 1	The men are given a pedometer and encouraged to record their daily step count in their booklet – ongoing
Self-monitoring of outcome of behaviour	Establish a method for the person to monitor and record the outcomes of the behaviour(s) as part of a behaviour change strategy	week 1-12, email 1, reunion	The men are encouraged to weigh themselves and record their weight in their booklet – ongoing
***Repetition and substitution***			
Behavioural practice/rehearsal	Prompt practice or rehearsal of the performance of the behaviour one or more times in a context or at a time when the performance may not be necessary, in order to increase habit and skill	week 2-12, reunion	The coach leads the men in physical activity sessions at the club each week and teaches them aerobic, strength and flexibility exercises they can do elsewhere – ongoing from week 2
Habit formation	Prompt rehearsal and repetition of the behaviour in the same context repeatedly so that the context elicits the behaviour	week 3-12	The men discuss how to increase their walking in their normal daily routines (week 3) and use this information to achieve their daily step count targets – ongoing from week 3
Behaviour substitution	Prompt rehearsal and repetition of an alternative behaviour to replace an unwanted habitual behaviour	week 3	The coach asks the men for examples of compensatory behaviour and for suggestions about how to avoid compensation – week 3
Generalisation of a target behaviour	Advise to perform a behaviour already performed in a particular situation, in another situation	week 4-8,11	The coach leads a discussion about local physical activity opportunities, provides a “Local Amenities Handout”, tells the men about any discounted offers and encourages those who are keen to set a SMART goal to try a new activity for next week – week 4
Graded tasks	Set easy-to-perform tasks, making them increasingly difficult, but achievable, until the behaviour is performed	week 2-12	The men set weekly step count targets that increase in difficulty as the programme progresses – ongoing from week 2
***Antecedents***			
Avoidance/reducing exposure to cues for the behaviour	Advise on how to avoid exposure to specific social and contextual/physical cues for the behaviour	week 2,3,10	The men identify triggers for eating, drinking and exercising, and the coach asks them to suggest strategies to avoid them – week 10
Adding objects to the environment	Add objects to the environment in order to facilitate performance of the behaviour	week 1	The men are given a pedometer to encourage them to be more active on a daily basis – week 1
Restructuring the social environment	Change, or advise to change the social environment in order to facilitate the behaviour or create barriers to the behaviour	week 3	The men are advised to plan to do something active with friends to avoid sitting in front of the TV – week 3
***Shaping knowledge***			
Information about antecedents	Provide information about antecedents that reliably predict performance of the behaviour	week 1,4,10	The coach asks the men to call out things that influence what they eat and leads a discussion about how difficult it can be to eat healthily and have an active life amidst all these other factors – week 1
Re-attribution	Elicit perceived causes of behaviour and suggest alternative explanations	week 1,11	The men put their initials on the “Locus of Control” (of eating) line in week 1 and the coach asks some to say why they placed their initials where they did. This exercise is repeated in week 11 and the men discuss how and why they have changed. The coach asks men who are still lower down the ‘scale’ what would help them move up the ‘scale’ and tries to identify some of the barriers stopping them doing this
Instruction on how to perform a behaviour	Advise or agree on how to perform the behaviour	week 1-11, email 6, reunion	The coach gives out pedometers, explains how they work and demonstrates the correct positioning of them on the body – week 1
***Self-belief***			
Focus on past successes	Advise to think about or list previous successes in performing the behaviour (or parts of it)	email 5	Email 5 suggests that now is a good time for the men to think back to what things were like before they started FFIT. What changes have they made to their eating and exercise routines since being on FFIT? How many of their old, unhealthy habits have they managed to replace by new, healthy eating and exercise habits?
Verbal persuasion about capability	Tell the person that they can successfully perform the behaviour, arguing against self-doubts and asserting that they can and will succeed	week 2,12, email 5, reunion	The coach tells the men how confident he is that they will be successful in their goals – reunion
***Goals and planning***			
Goal setting (behaviour)	Set or agree a goal defined in terms of the behaviour to be achieved	week 2-12, email 1,3-6, reunion	The coach asks the men to think back to the discussion of step-by-step changes the previous week, and to set new SMART goals for where they want to be in 1 month’s time – week 12
Goal setting (outcome)	Set or agree a goal defined in terms of a positive outcome of wanted behaviour	week 3, email 1,3,4	The coach encourages the men to note down their own 5 and 10% weight loss targets on their “Personal Weekly Progress Record”. He asks them how they feel about these figures and whether they are achievable – week 3
Behavioural contract	Create a written specification of the behaviour to be performed, agreed by the person and witnessed by another	week 2	The coach asks the men in pairs to think about their food diaries, discuss two goals to help them eat more healthily and write them in the “Setting SMART Goals Week 2” boxes – week 2
Commitment	Ask the person to make statements indicating strong commitment to change the behaviour	week 1	The coach stresses that he needs full commitment to the programme from everyone and that nobody should have more than 2 absences. He asks if anyone can foresee any difficulties with that – week 1
Action planning (includes Implementation intentions)	Prompt detailed planning of performance of the behaviour (must include at least one of context, frequency, duration and intensity)	week 2-5,7,10,12, email 2, reunion	The men are told their goals must be SMART: Specific, Measurable, Achievable, Recorded and Time limited – week 2
Review behaviour goals	Review behaviour goal(s) jointly with the person and consider modifying goal(s) or behaviour change strategy in light of achievement	week 3-8,10,12	The coach asks the men to spend 5 minutes in their teams discussing their goals from last week and setting new ones using the “Setting SMART Goals Week 5” boxes – week 5
Review outcome goal(s)	Review outcome goal(s) jointly with the person and modify goal(s) or behaviour change strategy in light of achievement	week 6,7,12	The coach asks the men to compare their 5 and 10% weight loss targets with the weight they have lost so far, and to discuss in their teams whether they feel they are on course to achieving a 5-10% weight loss. If so, how are they doing this? If not, do they feel they could change anything? – week 7
Discrepancy between current behaviour and goal	Draw attention to discrepancies between a person’s current behaviour and the person’s previously set outcome goals, behavioural goals or action plans	week 3,7,12, email 2,3	The coach asks the men to look at their Week 2 SMART goals and encourages them to say what has gone well for them since the last session. He also asks if anything has gone less well; and, where goals have not been achieved, he asks the men to consider whether they were too ambitious – week 3
Problem solving (includes Relapse prevention)	Analyse factors influencing the behaviour, and generate or select strategies that include overcoming barriers and/or increasing facilitators	week 3-8,10-12, email 1,5, reunion	The coach encourages the men to talk about any problems they have had and how they have overcome them. If any men are currently experiencing problems, he encourages the other group members to provide suggestions about how they can overcome the difficulties – reunion
***Comparison of outcomes***			
Persuasive source	Present verbal or visual communication from a credible source in favour of or against the behaviour	week 5,6, email 5,6	The coach invites a guest (usually a former participant) to talk to the men about their experiences of the programme – week 6
***Identity***			
Framing/reframing	Suggest the deliberate adoption of a perspective or new perspective on behaviour in order to change cognitions or emotions about performing the behaviour	week 11	Men repeat the “Locus of Control” (of eating) exercise, and discuss how and why this has changed since the first session. The coach asks the men who are still lower down the ‘scale’ what would help them move up the ‘scale’ and tries to identify some of the barriers stopping them doing this – week 11
***Natural consequences***			
Information about health consequences	Provide information about the health consequences of performing the behaviour	week 3,4, email 3	The coach tells the men how being physically active is associated with reducing risk of heart disease, reducing risk of cancer and avoiding depression – week 4
Information about emotional consequences	Provide information about the emotionalconsequences of performing the behaviour	week 3,4	The coach tells the men how losing just 5-10% of their starting weight and keeping it off can make them feel more alert and energetic, and improve their self-esteem and general outlook on life – week 3
Salience of consequences	Use methods to emphasise the consequences of changing the behaviour	week 7,11,12	Sand bags (or other tangible means of demonstrating weight loss both individually and in a group) are provided – week 7
Monitoring of emotional consequences	Prompt assessment of feelings after attempts at performing the behaviour	week 5,11	The coach asks the men to cast their mind back to the very first session and remember how they felt, and to compare that with how they feel right now and discuss any interesting differences – week 11
***Comparison of behaviour***			
Social comparison	Draw attention to others’ performance to explicitly elicit comparisons	week 5,7	The coach asks the men who are doing well increasing their physical activity how they feel as a result, in the hope that their positive feedback will inspire and encourage others – week 5
Demonstration of the behaviour	Provide an example of the behaviour being performed for the person to aspire to or imitate	week 2-12, reunion	The coach demonstrates aerobic, strength and flexibility exercises in club-based physical activity sessions – ongoing from week 2
***Covert learning***			
Vicarious consequences	Prompt observation of the consequences for others when they perform the behaviour	week 6	The coach invites a guest (usually a former participant) to talk to the men about their experiences of the programme – week 6

## Discussion

FFIT has been carefully developed to reflect: current best-practice weight management guidance [40,41]; an acceptable and valued delivery setting; and behaviour change techniques that are effective for weight loss, physical activity and healthy eating [56,57]. Care has been taken in the design of the programme and training materials to ensure that FFIT has potential to be readily generalisable and that fidelity of delivery is maximised [59], whilst enabling club coaches to feel a sense of ownership because they can draw on their own expertise, experience and style in delivering FFIT’s key messages. The multidisciplinary programme development working group had the theoretical, clinical and football club expertise to ensure that FFIT was grounded both theoretically and in its context of delivery. An iterative process was used in programme development, which allowed many potential strengths and weaknesses to be identified and addressed in the optimized (FFIT) delivery protocol. Finally, specification of the specific behaviour change techniques used, the intensity, duration and mode of delivery, and details about programme content, as recommended by WIDER^c^, makes it more likely that FFIT can be replicated [60].

### Limitations

FFIT draws on behaviour change techniques that are associated with a number of theoretical approaches and therefore could be criticised for lack of theoretical purity. However, a recent review suggests that weight loss, physical activity and healthy eating interventions that have an explicitly-stated theoretical basis are no more effective than those that do not [57]. Instead, FFIT uses well-defined behaviour change techniques, is based closely on existing evidence-based weight management and physical activity interventions [17,50], adheres to current weight management and physical activity guidance [40,41,46], and is cognisant of sociological understandings of the links between masculinities and health.

We did not include any members of our target population or SPL coaches in our programme development working group, although we actively sought and incorporated their feedback at all stages. Having end-user and coach representatives may have had benefits, including identification of some of the issues (e.g., practical constraints at the clubs) at an earlier stage.

Most of the clubs recruited participants through website and match day advertising; therefore, as these methods of recruitment do not permit estimation of the numbers of eligible men who were invited to participate, we are unable to report response rates. The recruitment procedures were monitored in the two clubs involved in the feasibility trial, and whilst we are still unable to estimate intervention reach [61], we report on the response to each recruitment strategy in these clubs elsewhere (Gray et al.: Can professional football clubs attract overweight men to a weight loss programme? A pilot randomised trial).

Time constraints meant that only a single session could be observed in most (9/11) clubs. Therefore whilst overall impressions of programme delivery were formed at each club, some club-specific issues with individual sessions may not have been identified. However, many sessions, particularly the key early sessions, were observed in at least two clubs, allowing some generalisations to be made. Feedback forms were only received from 51.2% of participants: nevertheless, the responses were consistent with the information obtained from the participant and coach focus groups and interviews, the coach feedback workshop and the programme session observations. Changes to the programme drew on all available sources of evidence.

Our age range meant that younger (18-34 years) men were excluded from taking part in p-FFIT. The lower age limit helped to foster a sense of belonging among group members who clearly appreciated being with men they viewed as similar to themselves. However, it is likely that younger men, who are also hard to engage in weight management, will be equally attracted to the professional football club setting, and work is now underway to explore the feasibility of offering similar programmes to a younger age group. Finally, job and family commitments were the most common reasons for a minority of participants being unable to complete p-FFIT. Future research developing online modules or mobile technological support (e.g., mobile phone applications) may help to address this issue.

## Conclusions

We have developed a programme that is highly acceptable to participants (overweight and obese men aged 35-65 years) and coaches at professional football clubs across Scotland. It is based on existing evidence-based weight loss and physical activity programmes, includes the key behaviour change techniques that are effective for weight loss, physical activity and healthy eating, and incorporates sociological understandings of men’s health and gender. The optimized programme (FFIT) is being evaluated in a pragmatic randomised controlled trial at all SPL clubs across Scotland [62]. This trial is funded by the National Institute for Health Research. The FFIT delivery manual is available on request from the corresponding author.

## Endnotes

^a^Programme delivery was funded by the Scottish Government and the Football Pools.

^b^http://www.spl-ffit.co.uk/page/daily-energy-requirements/

^c^Workgroup for Intervention Development and Evaluation Research.

## Competing interests

The authors declare that they have no competing interests.

## Authors’ contributions

CMG, NM, JL, ASA, KH and SW were members of the multidisciplinary programme development working group that designed and optimized the intervention. CMG conducted the focus group discussions, interviews and observations, performed the frequency analyses and drafted the manuscript. LD performed the qualitative analyses with support from CMG, who cross-coded three transcripts. All authors commented on drafts, and read and approved the final manuscript.

## Pre-publication history

The pre-publication history for this paper can be accessed here:

http://www.biomedcentral.com/1471-2458/13/232/prepub
